# Trends in domain-specific physical activity and sedentary behaviors among Chinese school children, 2004–2011

**DOI:** 10.1186/s12966-017-0598-4

**Published:** 2017-10-23

**Authors:** Tracy Dearth-Wesley, Annie Green Howard, Huijun Wang, Bing Zhang, Barry M. Popkin

**Affiliations:** 1 Department of Nutrition, Gillings School of Global Public Health, University of North Carolina at Chapel Hill, 137 E Franklin St, Room 6305, Chapel Hill, NC 27516 USA; 2 Carolina Population Center, University of North Carolina at Chapel Hill, Chapel Hill, NC USA; 3 Department of Biostatistics, Gillings School of Global Public Health, University of North Carolina at Chapel Hill, Chapel Hill, NC USA; 4 National Institute for Nutrition and Health, China Center for Disease Control and Prevention, Beijing, China

**Keywords:** China, Children, Adolescents, Physical activity, Sedentary, Trends

## Abstract

**Background:**

Dramatic increases in child overweight have occurred in China. A comprehensive look at trends in physical activity and sedentary behaviors among Chinese youth is needed. The study aimed to examine trends in domain-specific physical activity and sedentary behaviors, explore mean and distributional changes in predicted behaviors over time, and investigate how behaviors vary by residence.

**Methods:**

Using 2004–2011 China Health and Nutrition Survey data, adjusted means for MET-hours/week from physical activity and hours/week from sedentary behaviors were determined for school children (6–18 years), stratifying by gender, age group, and residence. Physical activity domains included in-school physical activity, active leisure (out-of-school physical activity), active travel (walking or biking), and domestic activity (cooking, cleaning, and child care). For each physical activity domain, the MET-hours/week measure was determined from the total weekly time spent (hours) in domain-specific activities and corresponding MET-values using the Compendium of Energy Expenditures for Youth. Sedentary behaviors included television, computer use, homework, and other behaviors (board games, toys, extracurricular reading and writing). For each sedentary behavior, the hours/week measure was determined from total weekly time spent in specific sedentary behaviors. Residence groups included megacities (population ≥ 20million), cities/towns (300,000 ≤ population < 20million), and rural/suburban areas (population < 300,000). Repeated measure linear mixed and quantile regression models were used to predict adjusted means.

**Results:**

Little change in physical activity behaviors occurred over time, with the exception of statistically significant trends toward increased domestic activity among male children (*p* < .05). Across all gender and age groups, statistically significant trends over time toward an average increase in computer use were seen (*p* < .01); these increases were largely driven by those ≥50th percentile on the distribution. Children living in megacities (versus rural areas) reported higher levels of physical activity, homework, and computer use.

**Conclusions:**

Intensified, systematic intervention and policy efforts promoting physical activity and reducing sedentary behaviors among children are needed.

**Electronic supplementary material:**

The online version of this article (10.1186/s12966-017-0598-4) contains supplementary material, which is available to authorized users.

## Background

China has experienced dramatic increases in child overweight over the 1991–2011 period, with much of these increases occurring between 2000 and 2011 [1]. The overweight prevalence in Chinese children (2–18 years) was 6.2% in 1991, 8.5% in 2000, and 15.4% in 2011 [1]. These increases are far greater proportionally than those overweight increases seen among Chinese adults [1, 2] and underscore the need for understanding corresponding changes in dietary and activity patterns. Studies have documented shifts in physical activity (PA) over the past couple decades among Chinese adults, revealing decreases in occupational and domestic (cooking, cleaning, and child care) PA and increases in sedentary behaviors [1, 3–7], but similar analyses have not been undertaken among Chinese children and adolescents. Short-term studies suggest limited PA at home or school and increased sedentary behavior, with significant gender and age differences [8–15]. However, a comprehensive look at more long-term, domain-specific trends in PA and sedentary behaviors among Chinese children and adolescents in recent years is lacking.

Understanding these domain-specific trends alongside research showing increases in child overweight [1, 16] and within the context of China’s rapidly changing social and economic environment [17, 18] is critical to informing obesity prevention policies and interventions. These changes and projected increases in overweight and obesity-related chronic diseases among Chinese children will continue to be detrimental to the population’s health and country’s productivity and economy [1, 19]. Exploration of trends in domain-specific PA and sedentary behaviors by gender, age, and residence (megacities, cities/towns, and rural/suburban areas) differentials and examination of changes in these behaviors across the distribution will provide new insight and enable future research to examine how policies or interventions are affecting specific populations.

Using data from the China Health and Nutrition Survey (CHNS), we examined PA and sedentary behaviors in Chinese school children (6–18 years) over a 7-year time period (2004–2011). PA behaviors included 4 domains (in-school, active leisure, active travel, and domestic), and sedentary behaviors also included 4 domains (homework, television, computer use, and other activities, such as board games and extracurricular reading). Our primary study objectives were to (1) examine trends in domain-specific PA and sedentary behaviors by gender and age group differentials, (2) explore changes in predicted sedentary behaviors over time at the mean and across the distribution, and (3) investigate how PA and sedentary behaviors vary among Chinese children living in megacities, cities/towns, and rural/suburban areas.

## Methods

### Data and subjects

The CHNS began in 1989 and utilized a multistage randomized cluster design drawing from 9 Chinese provinces that vary in geography, socioeconomic growth, and health indicators. Counties in these 9 provinces were stratified by income (low, middle, high); application of a weighted sampling scheme resulted in four randomly selected counties in each province. Random selection of villages, townships, and urban/suburban neighborhoods in each county was done. A total of 216 primary sampling units are included in CHNS surveys since 2000 (36 urban neighborhoods, 36 suburban neighborhoods, 36 towns, and 108 villages), which include roughly 4400 households and 26,000 individuals. Surveys were conducted every 2–4 years; in 2011, three megacities (Beijing, Shanghai, and Chongqing) were added to the sample [20]. Longitudinal CHNS data from surveys conducted in 2004, 2006, 2009 and 2011 was utilized as prior to that detailed physical activity data was not collected for children. The study was approved by the Institutional Review Boards of the University of North Carolina at Chapel Hill and the National Institute of Nutrition and Food Safety, China Center for Disease Control and Prevention.

Trained field staff collected survey data using structured questionnaires administered to all household members. Parents or primary caregivers helped complete questionnaires for children <10 years of age. Our study sample included children between the ages of 6 and 18 years who were in school. Additional CHNS details are available in previous publications [18, 20].

### Measures

#### PA behaviors

The PA outcome measures included total metabolic equivalent of task (MET)-hours per week in four activity domains: (1) in-school PA, (2) active leisure (out-of-school PA), (3) active travel, and (4) domestic PA. For each domain, the MET-hours per week measure was determined from the total weekly time spent (hours) in domain-specific activities and the corresponding MET-values for these activities using the Compendium of Energy Expenditures for Youth [11, 21]. Children were asked about their participation and time spent in specific activities within each domain.

For the in-school PA and active leisure domains, children were asked about their participation in 6 activity categories: (1) martial arts, (2) gymnastics, dancing, acrobatics, (3) track and field, swimming, (4) soccer, basketball, tennis, (5) badminton, volleyball, and (6) other (ping pong, Tai Chi, etc.). Average MET-values for each activity category were determined. For in-school PA, children reported on their weekly time spent in each activity category. For active leisure, children reported on time spent before or after school during a typical weekday and weekend day. Weekly time spent in active leisure activities was determined by summing the following products: typical weekday time × 5; typical weekend day time × 2. Weekly time spent in each activity category was multiplied by the corresponding average MET-value and summed to determine the total MET-hours per week for the in-school PA and active leisure domains.

For the active travel domain, children were asked about their participation and time spent walking and/or pedaling a bike to and from school each day. Weekly time spent in each activity was determined by multiplying the daily time spent by 5. The weekly time spent in each activity was then multiplied by the corresponding MET-value and summed for the total MET-hours per week for active travel. For the domestic activity domain, children were asked about their participation and time spent per day in the following activities: buying and cooking food, doing laundry, cleaning the house, and caring for children. Weekly time spent in each activity was determined by multiplying the daily time spent by 7. The weekly time spent in each activity was then multiplied by the corresponding MET-value and summed for the total MET-hours per week for domestic activity.

#### *Sedentary behavior*s

The sedentary behavior outcome measures included total hours per week from four sedentary behavior categories: (1) television, (2) computer use, (3) homework, and (4) other. For each category, children were asked about their participation and time spent in specific activities on a typical weekday and weekend day. Weekly time spent in each activity was determined by summing the following products: typical weekday time × 5; typical weekend day time × 2. Activities in the television category included watching TV, video games, videotapes, VCDs, DVDs, and movies/videos online. Computer use activities included surfing the Internet, participating in chat rooms, and playing computer games. Children reported on homework time in a separate question, and this homework measure did not include computer time (e.g., surfing the Internet) for homework purposes. Activities in the other category included playing with board games, toy cars, and puppets and extracurricular reading, writing, and drawing.

#### Covariates

Additional variables used in the analyses included child age and gender, highest level of maternal education, wave-specific income tertiles, urbanicity index, and residence. The urbanicity index is a continuous measure that includes 12 dimensions of modernization: population density, economic activity, traditional markets, modern markets, transportation infrastructure, sanitation, communications, housing, education, diversity, health infrastructure, and social services [20, 22]. Residence groupings include megacities (population ≥ 20million), cities/towns (300,000 ≤ population < 20million), and rural/suburban areas (population < 300,000).

### Analysis

Trends in average MET-hrs/wk from PA (in-school, active leisure, active travel, and domestic) and average hrs/wk from sedentary behaviors (homework, television, computer, and other) were determined for children in 2004, 2006, 2009, and 2011. Means were predicted from repeated measures linear mixed models and were adjusted for wave-specific income tertile, urbanicity index, maternal education, and child age. Analyses were stratified by gender and age group (6–11 y; 12–18 y), and megacities’ data in CHNS 2011 was not included. Post-hoc tests of linear trends were done to examine changes in PA or sedentary behaviors over time.

Predicted hours per week (hrs/wk) from sedentary behaviors (homework, television, computer, and other) were determined using quantile regression (QR) models in addition to the mean predicted values from the linear mixed models mentioned above. QR models were used to better understand the patterns of change seen in sedentary behaviors, particularly at the upper end of the distribution. QR models reported on the 50th, 75th, and 90th percentiles and yield additional information for understanding what is driving mean changes in sedentary behavior over time. Predicted hrs/wk in sedentary behaviors were determined separately by gender and age group; separate QR models were done in 2004 and 2011. All models adjusted for wave-specific income tertile, urbanicity index, maternal education, and child age.

Inclusion of the megacities data in CHNS 2011 allowed for examination of PA and sedentary behaviors by residence groupings: megacities, cities/towns, and rural/suburban areas (the latter two residence groups found within provinces) using models restricted to 2011 data only. Average values for PA (MET-hrs/wk) and sedentary (hrs/wk) behaviors were predicted from ordinary least squares (OLS) models, as we had no repeated data collected for the megacities. The models adjusted for income tertile, maternal education, and child age and were stratified by gender and age group. The Kruskall-Wallis test was used to examine differences in predicted PA and sedentary behaviors in children in 2011 from the OLS models across the three groupings (megacities, cities/towns, and rural/suburban areas). Subsequent pairwise comparison of groups using the Dunn test was conducted to determine if there were significant pairwise differences. All analyses were conducted using Stata version 14.0 (Stata Corporation, College Station, TX, USA).

## Results

Descriptive statistics for Chinese school children by age group across the 4 survey years are shown in Table 1. Among children in both age groups, just under half were female across the survey years. Increases in the urbanization index and level of maternal education over the 7 years were seen among children in both age groups.

**Table 1:** Descriptive statistics for Chinese school children by age group, China Health and Nutrition Survey (CHNS) 2004-2011^a^

	CHNS Year			
	2004	2006	2009	2011
Children, 6–11 y				
N	722	687	628	629
Female, %	47.2	46.7	42.2	48.3
Age, years (SD)	9.2 (1.7)	9.3 (1.6)	9.2 (1.7)	9.1 (1.7)
Urbanization index (SD)	57.9 (19.4)	59.7 (19.6)	63.0 (18.4)	66.0 (18.0)
Maternal education, %				
Less than primary	7.3	11.4	9.8	8.0
Primary completed	30.2	19.6	23.0	18.4
Secondary or greater completed	62.5	68.9	67.2	73.6
Children, 12–18 y				
N	837	566	508	422
Female, %	48.3	48.1	47.4	48.1
Age, years (SD)	14.7 (1.6)	14.7 (1.7)	14.3 (1.6)	14.5 (1.6)
Urbanization index (SD)	61.7 (20.0)	65.1 (20.3)	67.4 (19.4)	70.7 (18.5)
Maternal education, %				
Less than primary	15.5	12.2	14.4	10.5
Primary completed	21.0	16.5	23.6	19.9
Secondary or greater completed	63.6	71.3	62.0	69.6

^a^Megacities (Beijing, Shanghai, and Chongqing) added in CHNS 2011 are not included in Table. Percentages across levels of maternal education may not add to 100% due to rounding

### Physical activity and sedentary behavior trends

**Fig. 1:**
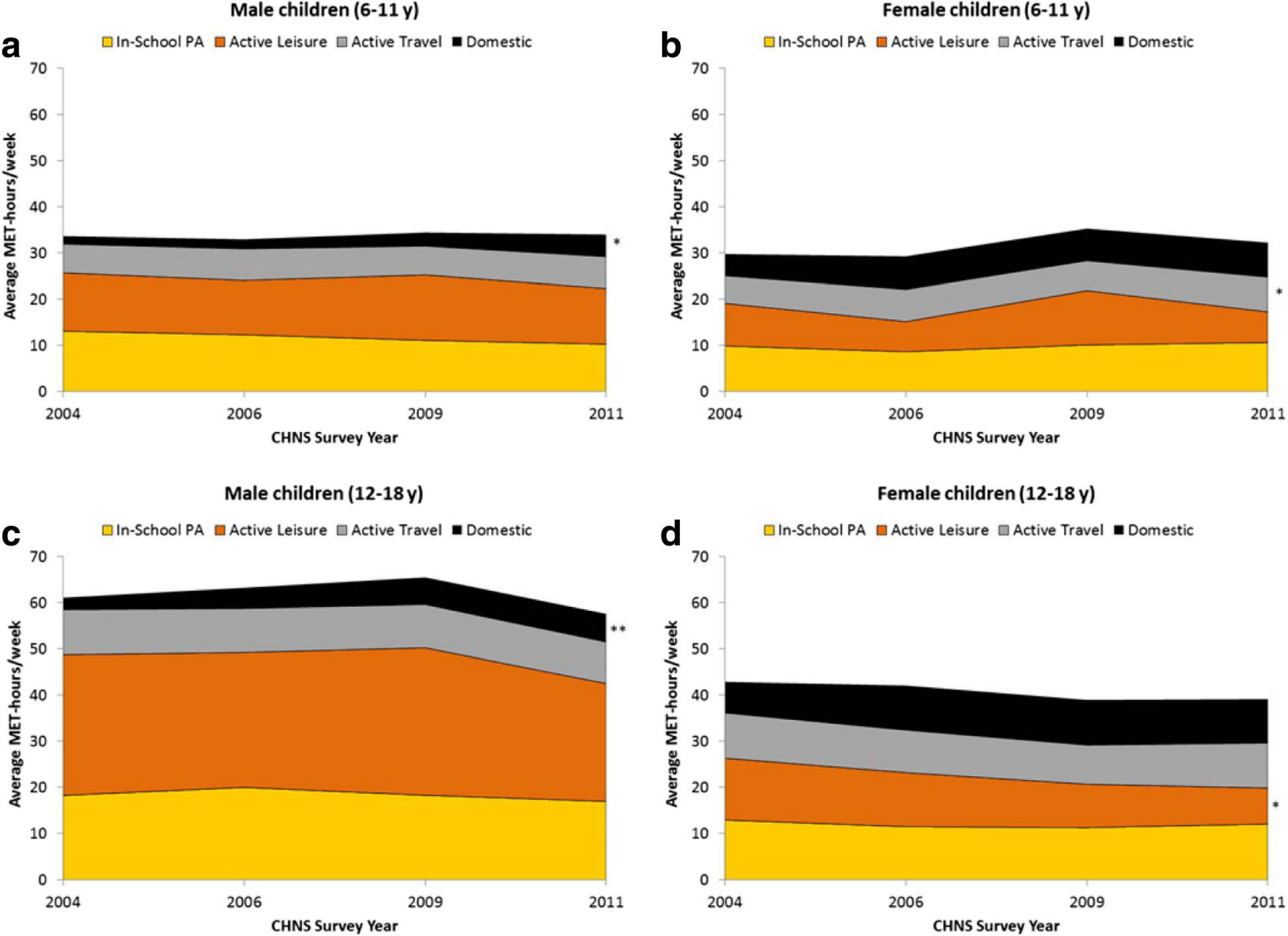
**a**-**d.** Trends in adjusted means for physical activity (MET-hours/week) in Chinese school children by gender and age group, China Health and Nutrition Survey (CHNS)^a^. ^a^ Means are adjusted for wave-specific income tertile, urbanicity index, maternal education, and child age. ^*^ *P*-value <.05 from post-hoc test of linear trend. ^**^ *P*-value <.001 from post-hoc test of linear trend

On average, PA (MET-hrs/wk) among male children (both age groups) is primarily comprised of in-school PA and active leisure, whereas PA behaviors among female children (both age groups) is more balanced across the four domains (Fig. 1). This pattern is consistent across the survey years. Little change is seen in most PA behaviors over time; however, a statistically significant trend toward increased domestic PA is seen among male children in both age groups (*p* < .05).

**Fig. 2:**
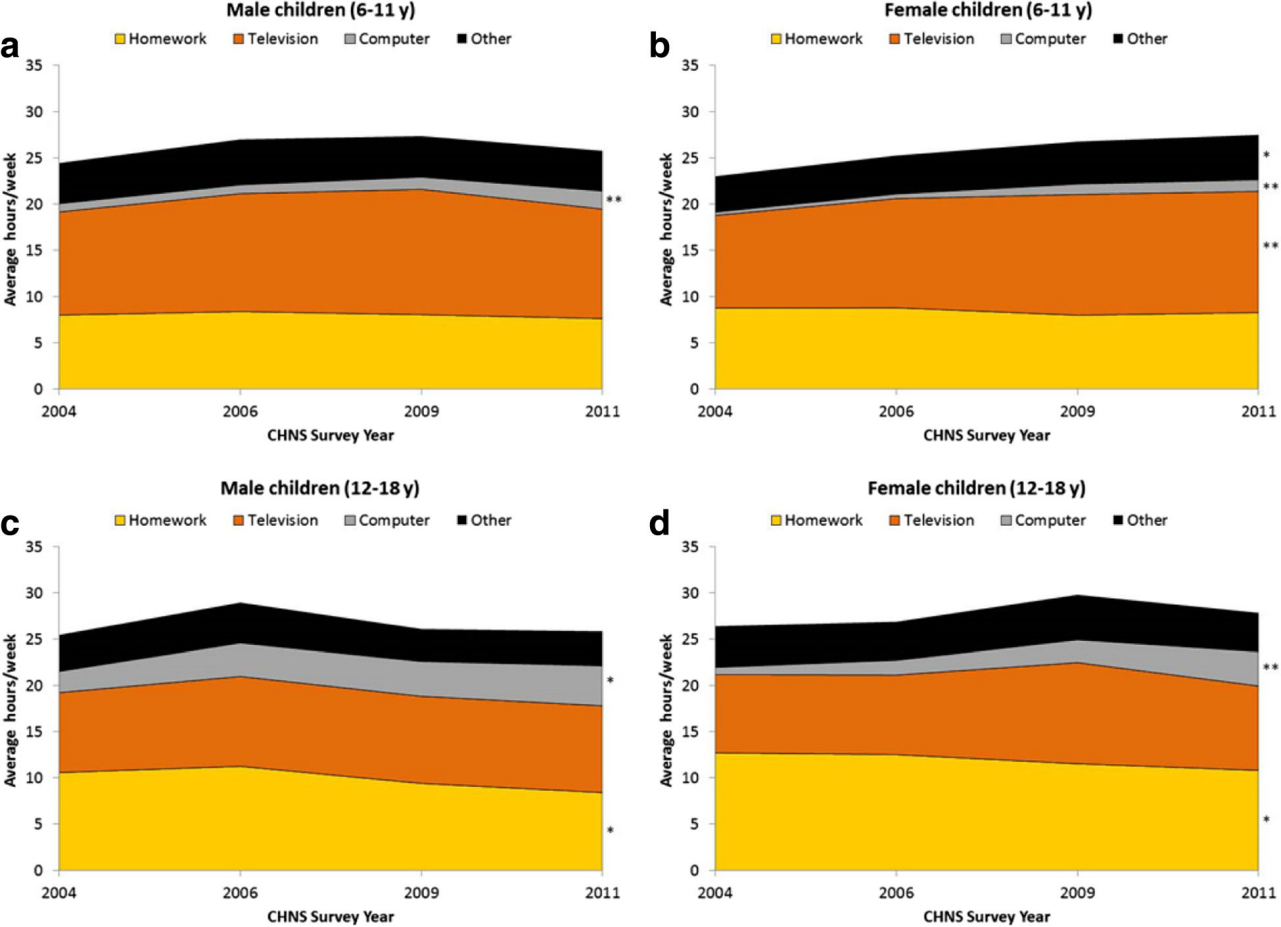
**a**-**d.** Trends in adjusted means for sedentary behaviors (hours/week) in Chinese school children by gender and age group, China Health and Nutrition Survey (CHNS)^a^. ^a^Means are adjusted for wave-specific income tertile, urbanicity index, maternal education, and child age. ^*^ *P*-value <.05 from post-hoc test of linear trend. ^**^ *P*-value <.001 from post-hoc test of linear trend

On average, television use, followed by homework time, are the predominant contributors toward sedentary behavior (hrs/wk) among male and female children (6–11 y) (Fig. 2). This pattern is consistent over time. For male and female children (12–18 y), homework is a larger contributor toward sedentary behavior than television use across the survey years. However, statistically significant trends toward decreased homework time are seen for male and female children (12–18 y) (*p* < .05). Across all gender and age groups, statistically significant trends toward increased computer use are seen (*p* < .01).

### Quantile regression: the distribution of trends

Examination of predicted sedentary behaviors (hrs/wk) using linear mixed models and QR models reveals that increases in computer use from 2004 to 2011 are largely driven by those in the 50th percentile or higher on the distribution (Table 2). For example, there was no computer use among male and female children (12–18 y) at the 50th percentile in 2004. In contrast, computer use among male and female children (12–18 y) at the 50th percentile in 2011 was 2.6 and 1.7 h/wk. Similarly, there was no computer use for male and female children (6–11 y) at the 75th percentile in 2004, yet computer use was 2.5 h/wk for male and 1.4 h/wk for female children (6–11 y) at the 75th percentile in 2011. The predicted 90th percentile for hrs/wk from computer use more than doubled across all gender and age groups from 2004 to 2011. Decreases over time in homework time were less distinct across the distribution, with lower predicted hrs/wk reported in 2011 vs 2004 across most of the percentiles for children by gender and age groups.

**Table 2:** Predicted sedentary behaviors (hours/week) in Chinese school children by gender and age group using linear mixed models and quantile regression (QR) Models, China Health and Nutrition Survey 2004 and 2011^a^

	Linear models		QR 50th		QR 75th		QR 90th	
	2004	2011	2004	2011	2004	2011	2004	2011
Male children, 6-11y								
Television	11.1 (10.3,12.0)	11.8 (10.9,12.8)	9.5 (8.5,10.4)	10.0 (8.9,11.2)	14.9 (13.4,16.4)	14.9 (13.6,16.3)	21.0 (19.0,22.1)	20.5 (16.5,24.5)
Computer	0.9 (0.5,1.3)	1.9 (1.5,2.4)	0.0 (0.0,0.0)	0.0 (0.0,0.0)	0.0 (0.0,0.0)	2.5 (1.6,3.3)	3.5 (2.0,5.1)	7.1 (5.2,8.9)
Homework	8.0 (7.3,8.8)	7.7 (6.8,8.5)	7.1 (6.5,7.7)	7.0 (6.3,7.7)	10.9 (9.8,12.0)	10.6 (9.4,11.7)	14.8 (13.1,16.4)	14.2 (11.7,16.7)
Other^b^	4.4 (3.8,5.1)	4.4 (3.7,5.1)	2.6 (2.0,3.1)	3.0 (2.2,3.7)	6.3 (5.2,7.3)	6.2 (5.2,7.3)	11.0 (9.2,12.7)	11.1 (8.4,13.7)
Female children, 6-11y								
Television	10.0 (9.1,10.9)	13.1 (12.1,14.1)	9.1 (8.2,10.0)	11.5 (10.1,12.9)	13.3 (12.0,14.5)	17.1 (15.7,18.6)	18.5 (15.5,21.5)	22.5 (18.9,26.1)
Computer	0.3 (0.1,0.6)	1.2 (0.9,1.6)	0.0 (0.0,0.0)	0.0 (0.0,0.0)	0.0 (0.0.,0.0)	1.4 (0.5,2.3)	0.0 (0.0,0.0)	4.5 (2.9,6.1)
Homework	8.8 (7.9,9.6)	8.4 (7.3,9.4)	7.8 (7.0,8.7)	7.1 (6.4,7.7)	11.5 (10.6,12.5)	10.4 (9.0,11.7)	15.4 (13.4,17.4)	15.7 (12.5,18.9)
Other^b^	3.9 (3.3,4.5)	4.9 (4.2,5.5)	2.6 (2.1,3.2)	3.8 (3.2,4.5)	5.5 (4.8,6.2)	6.8 (5.7,7.9)	7.6 (6.1,9.1)	11.1 (7.4,14.8)
Male children, 12-18y								
Television	8.7 (7.9,9.4)	9.2 (8.2,10.2)	7.3 (6.6,7.9)	7.7 (6.6,8.8)	12.1 (10.4,13.8)	11.9 (10.0,13.8)	19.0 (17.0,20.9)	18.8 (16.3,21.3)
Computer	2.3 (1.6,2.9)	4.3 (3.4,5.3)	0.0 (0.0,0.0)	2.6 (1.8,3.4)	2.3 (1.3,3.3)	6.5 (4.6,8.4)	6.2 (4.3,8.0)	14.4 (10.8,18.0)
Homework	10.6 (9.7,11.5)	8.4 (7.2,9.7)	8.5 (7.7,9.4)	8.1 (7.0,9.2)	14.5 (13.2,15.7)	12.1 (10.7,13.6)	21.4 (18.4,24.4)	16.7 (14.4,19.0)
Other^b^	3.9 (3.4,4.4)	3.7 (3.0,4.4)	3.1 (2.6,3.6)	3.0 (2.2,3.7)	5.3 (4.5,6.1)	6.1 (5.3,6.9)	9.3 (8.2,10.5)	8.9 (7.1,10.8)
Female children, 12-18y								
Television	8.5 (7.7,9.3)	9.2 (7.9,10.4)	7.1 (6.4,7.8)	7.4 (6.4,8.5)	11.0 (9.7,12.2)	12.3 (10.6,13.9)	18.5 (15.4,21.5)	17.9 (14.4,21.4)
Computer	0.8 (0.3,1.2)	3.7 (3.0,4.4)	0.0 (0.0,0.0)	1.7 (0.6,2.7)	0.0 (0.0,0.0)	5.8 (3.7,7.8)	2.7 (2.1,3.3)	12.8 (9.4,16.1)
Homework	12.7 (11.8,13.7)	10.8 (9.3,12.3)	10.6 (9.4,11.8)	10.0 (8.4,11.6)	17.2 (16.0,18.5)	15.6 (13.6,17.5)	21.6 (18.6,24.7)	21.3 (17.7,25.0)
Other^b^	4.4 (3.9,5.0)	4.2 (3.3,5.0)	3.5 (3.0,4.0)	3.4 (2.7,4.0)	6.2 (5.5,6.8)	5.9 (4.8,7.0)	9.2 (7.6,10.8)	8.7 (4.9,12.5)

^a^Predicted values control for wave-specific income tertile, urbanicity index, maternal education, and child age

^b^Other = board games + toys cars and puppets + extracurricular reading, writing and drawing

### PA and sedentary behaviors by area of residence

Lastly, examination of PA behaviors in 2011 by residence groupings found that children living in megacities reported the highest levels of PA (MET-hrs/wk), namely from active leisure and in-school PA (Additional file 1: Figure S1). For sedentary behaviors, children living in megacities reported the highest levels of homework time across all age and gender groups; these levels were statistically significantly different than those for children living in rural/suburban areas (*p* < .05) (Additional file 2: Figure S2). In contrast, children living in rural/suburban areas reported the highest levels of television across all age and gender group; these levels were statistically significantly different than those for children living in megacities (*p* < .001 for all age and gender groups except male children, 12-18y). Computer use for children living in megacities was statistically significantly higher than that for children in rural/suburban areas across both gender and age groups (*p* < .001).

## Discussion

Our study provides a comprehensive look at domain-specific trends for PA and sedentary behaviors among Chinese youth for the 2004–2011 period. With the exception of small increases in domestic PA among male children, there was little change in PA over time. In contrast, increases in sedentary behavior, namely in computer use, were seen. Our findings also suggest shifts away from homework toward computer or television time depending on the area of residence. Variations in domain-specific PA and sedentary behavior patterns were seen by gender and age group, with more similar patterns seen by gender for PA and by age group for sedentary behaviors.

While domestic PA has historically not been a part of daily activity among Chinese youth [8, 9], we documented statistically significant trends toward increased domestic PA among male children in both age groups. A decline in domestic PA among women in China over the past two decades, particularly at the higher percentiles [5], suggests that male Chinese children may be taking on additional domestic responsibilities. Despite these small changes in domestic PA, little to no change in other PA behaviors over time across gender and age groups is problematic. These PA findings coupled with China’s nutrition transition [17, 23] will continue to detrimentally impact the health of Chinese youth [19, 24] and demand intensified intervention and policy efforts.

Increases in computer time over the course of this study were seen among all gender and age groups, with distributional changes not only showing more children reporting computer use over time but also longer times reported at the higher percentiles. These findings along with differences in computer use by residence groupings are consistent with trends in computer ownership in Chinese households from 1999 to 2009, showing increases in the average number of computers per 100 households from 5.9 to 65.7 in urban households (1999–2009) and from 0.4 to 7.5 in rural households (2000–2009) [25]. Additionally, while the average daily computer use among older Chinese children is less than half of the 1.5 h/day reported among U.S. children [26], our distributional trend data suggests this difference will likely diminish in the coming years. Continual increases in computer use, relatively stable television use with some variation by residence, and minimal changes in PA all contribute to an increasingly sedentary Chinese youth population.

While continued policy and intervention efforts working to encourage activity and limit sedentary time are essential, our findings highlight the need for more targeted efforts that incorporate sociodemographic differences. Similarities in domain-specific PA behaviors were seen in male vs. female children, whereas similarities in domain-specific sedentary behaviors were found in younger vs. older children. These variations along with differences by residence suggest efforts, such as reducing television use among younger children (particularly among those living in rural/suburban areas) and promoting more active leisure among female children across both age groups, are important. Similar research has been done in other countries looking at correlates of PA and sedentary behaviors and trends, yielding similar support for more targeted interventions and policies [27–29]. Additionally, our research provides a platform for future study into how intervention and policy work affects domain-specific PA and sedentary behaviors among particular at-risk Chinese youth populations.

There are several study limitations that warrant explanation. First, the CHNS it is not a nationally representative survey, but previous findings strongly correlate with survey trends from the National Nutrition and Health Survey [30, 31]. Secondly, it is not possible based on the CHNS questions to determine if computer time (specifically surfing the Internet) was used for homework vs. leisure purposes. Computer use for homework purposes is not included in the homework measure and thus not accounted for in the declining trend in homework seen among older male and female children. Also, PA and sedentary behaviors are based on self-report (children ≥10 years) and parent-assisted self-report (children <10 years). While the parent-assisted self-report was employed to improve accuracy, varying data collection methods were used. Additionally, self-reported PA data may be subject to recall and social desirability biases. While survey questions were designed to facilitate the reporting of PA and sedentary behaviors in children, the questions did include various time frames (e.g., weekly time spent for in-school PA versus typical weekday and weekend day time for active leisure). While not subject to formal reliability and validity testing, studies using the CHNS PA questions have shown significant relationships between PA behaviors and health outcomes [4, 32–35]. Some of these relationships include increased occupational PA and lower body weight in men and women (beta-coefficient (95% CI): -0.46 (−0.76, −0.15) and −0.36 (−0.62, −0.10), respectively) [32]; decreased PA and 6.1% weight gain in men [34]; and moderate or heavy PA and lower systolic blood pressure in men and women [35]. Lastly, while the MET-hrs/wk measurement does not address individual differences in energy expenditure associated with self-reported PA, it remains an appropriate measure for estimating energy costs associated with self-reported PA [36].

## Conclusion

Our findings documenting an increasingly sedentary Chinese youth population correspond with dramatic increases in child overweight over the same time period [1]. These patterns alongside China’s nutrition transition present major, multifaceted public health challenges with numerous implications on the country’s health, productivity, and future. Early interventions and policies promoting PA, limiting sedentary behavior, and encouraging healthy eating are essential to the overall health and well-being of the Chinese population.

## Additional files

Additional file 1: Figure S1. **a-d**. Adjusted means for physical activity (MET-hours/week) in 2011 among Chinese school children by residence, gender, and age group, China Health and Nutrition Survey (CHNS)^a^. ^a^ Means are adjusted for income tertile, maternal education, and child age. (DOCX 752 kb)

Additional file 2: Figure S2. **a-d**. Adjusted means for sedentary behaviors (hours/week) in 2011 among Chinese school children by residence, gender, and age group, China Health and Nutrition Survey (CHNS)^a^. ^a^ Means are adjusted for income tertile, maternal education, and child age. ^*^ Homework (hrs/wk) statistically different between megacities and rural/suburban areas (*p* < .05). ^†^ Television (hrs/wk) statisctically different between megacities and rural/suburban areas (*p* < .001). ^‡^ Computer (hrs/wk) statistically different between megacities and rural/suburban areas (*p* < .001). (DOCX 767 kb)

## Abbreviations

CHNS: China Health and Nutrition Survey
MET: Metabolic equivalent of task
PA: Physical activity
