# Supplementary figures and images for: Trends in domain-specific physical activity and sedentary behaviors among Chinese school children, 2004–2011
Source: Int J Behav Nutr Phys Act. 2017 Oct 23;14:141. doi: 10.1186/s12966-017-0598-4 (PMC5651590; doi:10.1186/s12966-017-0598-4)

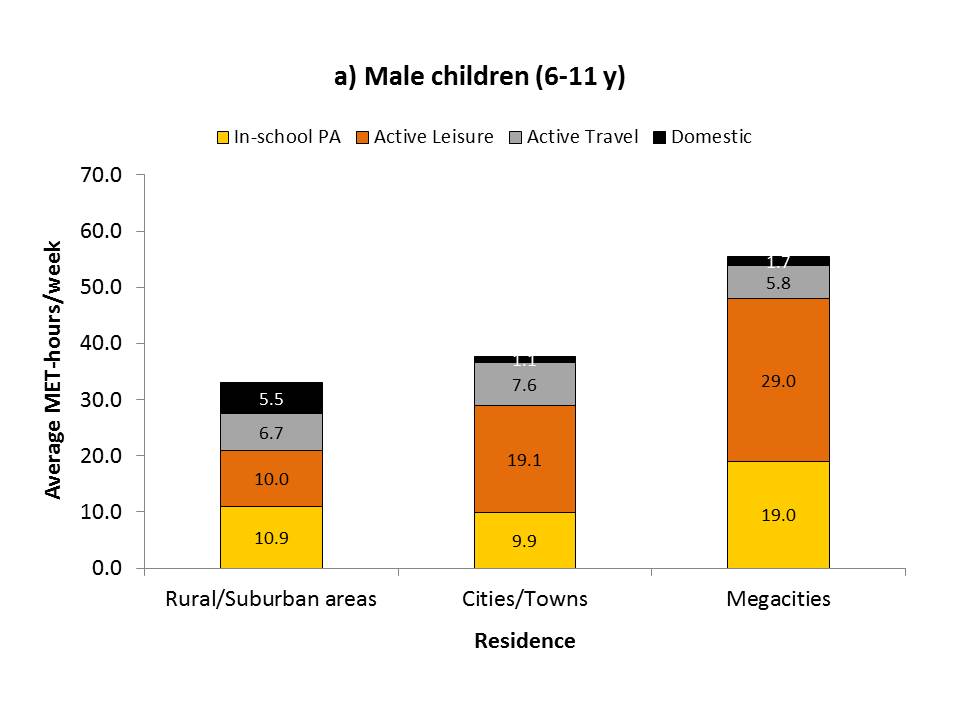

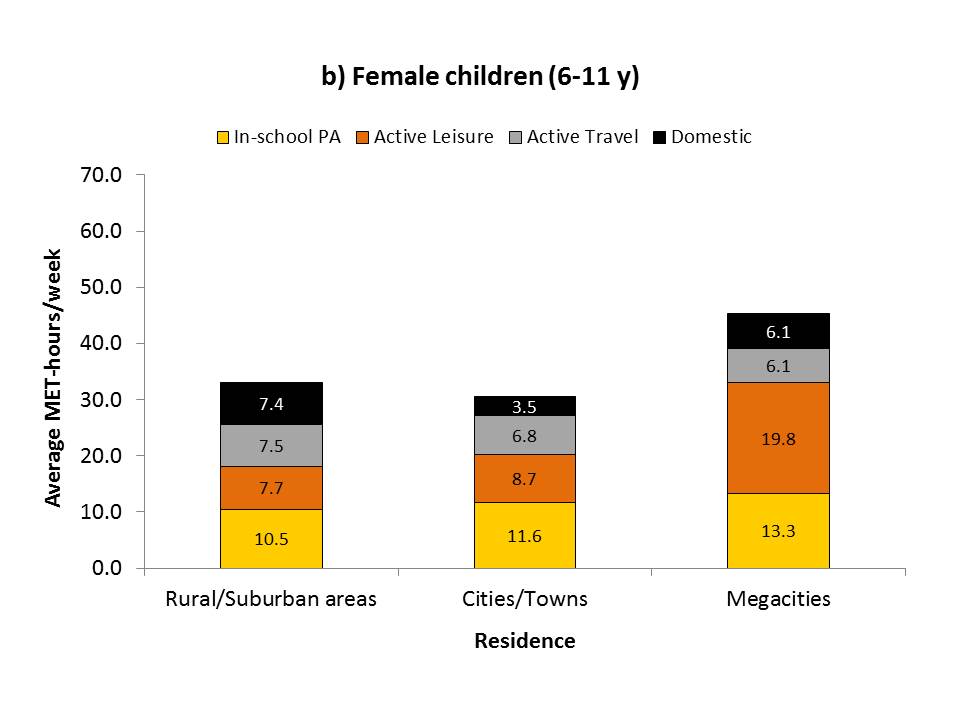

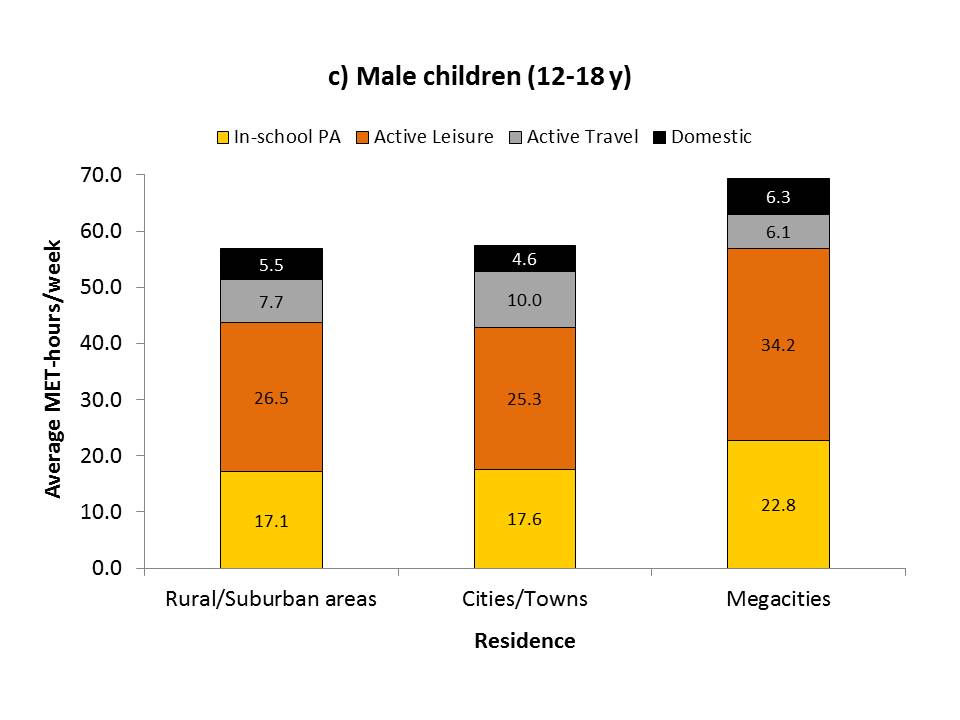

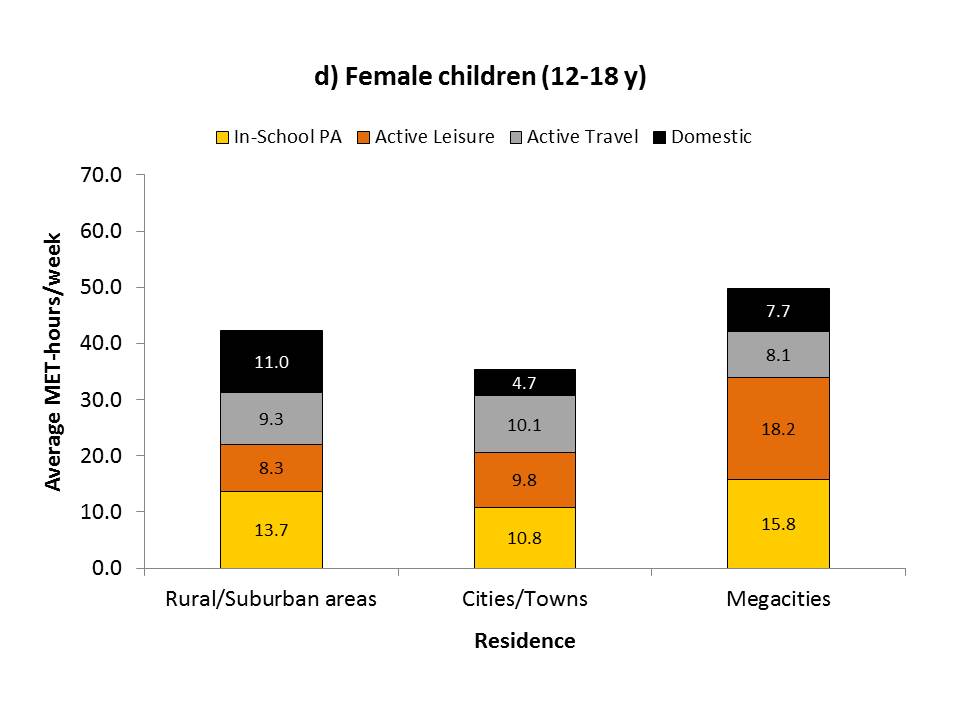

Supplement: Supplementary file 1 — a-d. Adjusted means for physical activity (MET-hours/week) in 2011 among Chinese school children by residence, gender, and age group, China Health and Nutrition Survey (CHNS)a. a Means are adjusted for income tertile, maternal education, and child age. (DOCX 752 kb) [file 12966_2017_598_MOESM1_ESM.docx]

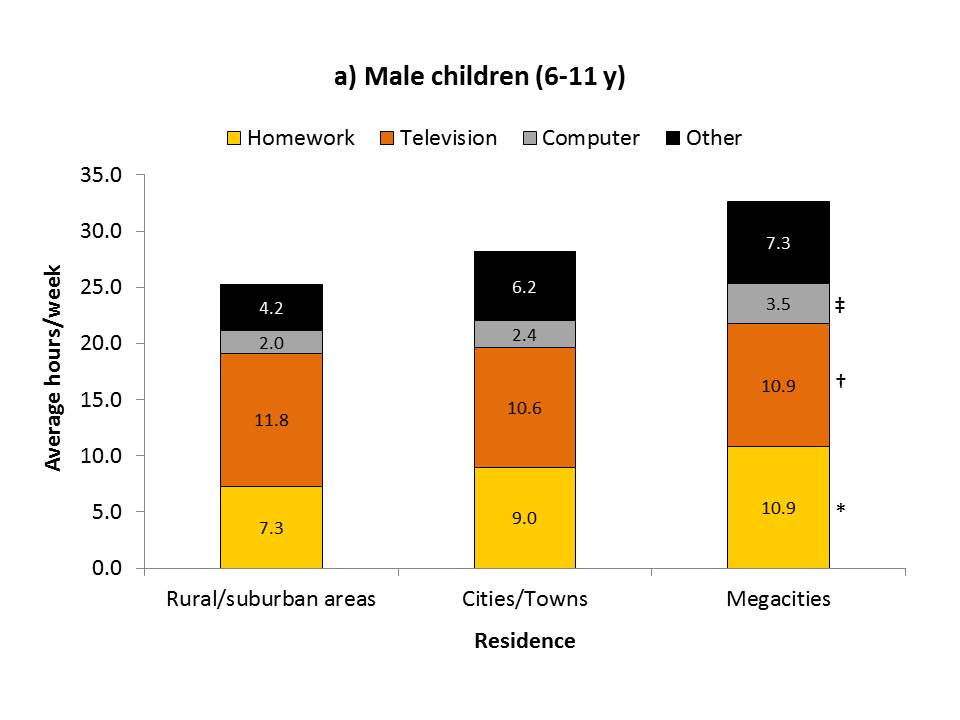

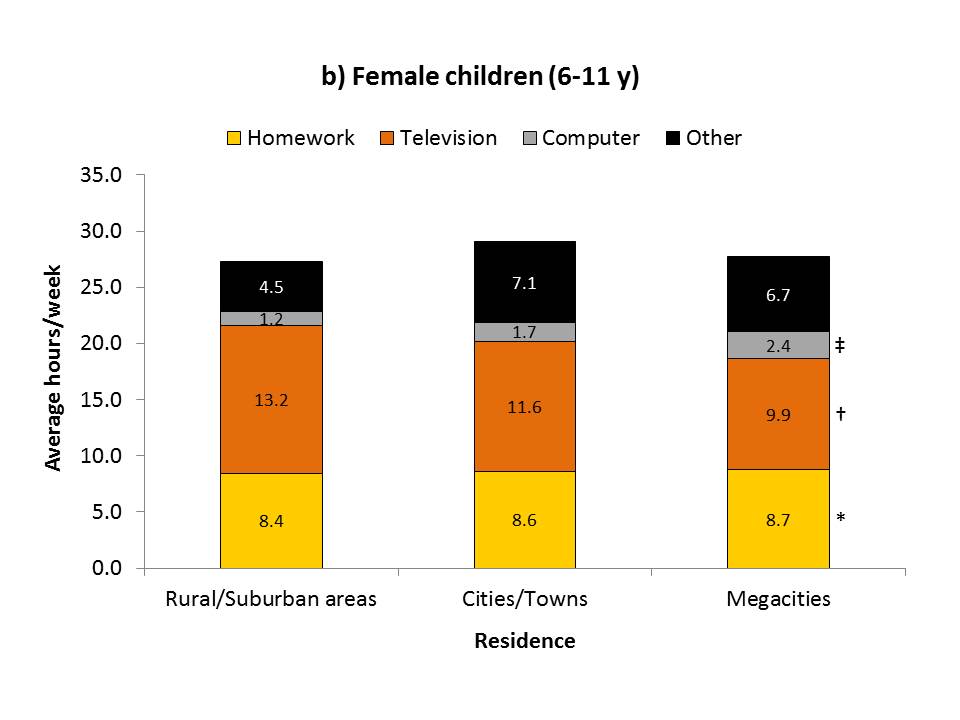

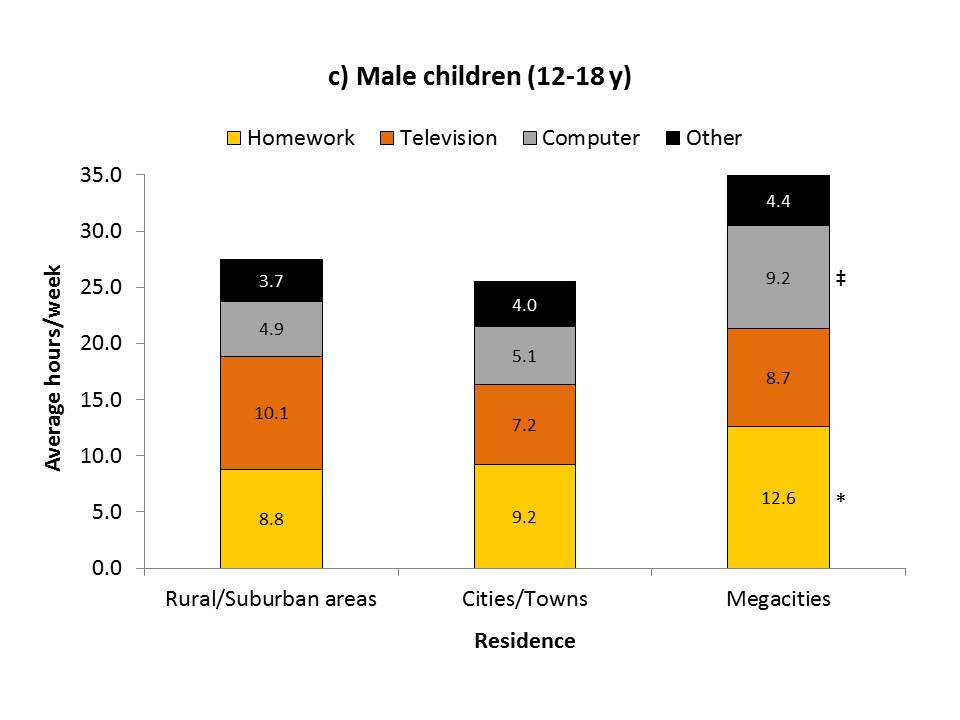

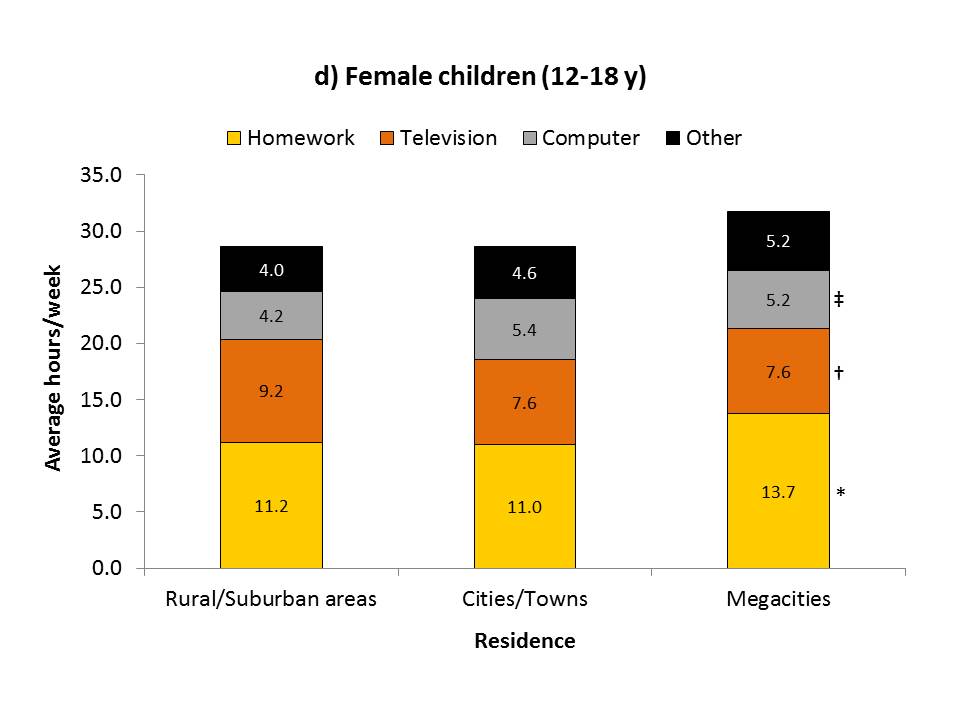

Supplement: Supplementary file 2 — a-d. Adjusted means for sedentary behaviors (hours/week) in 2011 among Chinese school children by residence, gender, and age group, China Health and Nutrition Survey (CHNS)a. a Means are adjusted for income tertile, maternal education, and child age. * Homework (hrs/wk) statistically different between megacities and rural/suburban areas (p < .05). † Television (hrs/wk) statisctically different between megacities and rural/suburban areas (p < .001). ‡ Computer (hrs/wk) statistically different between megacities and rural/suburban areas (p < .001). (DOCX 767 kb) [file 12966_2017_598_MOESM2_ESM.docx]
